# D-amino acids reduce *Enterococcus faecalis* biofilms *in vitro* and in the presence of antimicrobials used for root canal treatment

**DOI:** 10.1371/journal.pone.0170670

**Published:** 2017-02-02

**Authors:** Peter S. Zilm, Victor Butnejski, Giampiero Rossi-Fedele, Stephen P. Kidd, Suzanne Edwards, Krasimir Vasilev

**Affiliations:** 1 Microbiology laboratory, The School of Dentistry, The University of Adelaide, Adelaide, South Australia, Australia; 2 Australian Centre for Antimicrobial Resistance Ecology, Research Centre for Infectious Disease, School of Biological Science, The University of Adelaide, Adelaide, South Australia, Australia; 3 School of Public Health, The University of Adelaide, Adelaide, South Australia, Australia; 4 School of Engineering, University of South Australia, Adelaide, South Australia, Australia; University of Padova, Medical School, ITALY

## Abstract

*Enterococcus faecalis* is the most frequent species present in post-treatment disease and plays a significant role in persistent periapical infections following root canal treatment. Its ability to persist in stressful environments is *inter alia*, due to its ability to form biofilms. The presence of certain D-amino acids (DAAs) has previously been shown to reduce formation of *Bacillus subtilis* biofilms. The aims of this investigation were to determine if DAAs disrupt biofilms in early and late growth stages for clinical *E*. *faecalis* strains and to test their efficacy in disrupting *E*. *faecalis* biofilms grown in sub-minimum inhibitory concentrations of commonly used endodontic biocides. From thirty-seven *E*. *faecalis* strains, the ten “best” biofilm producers were used to test the ability of a mixture containing D-leucine, D-methionine, D-tyrosine and D-tryptophan to reduce biofilm growth over a period of 24, 72 and 144 hours and when compared to their cognate L-Amino Acids (LAAs). We have previously shown that sub-MIC levels of tetracycline and sodium hypochlorite promotes biofilm growth in clinical strains of *E*. *faecalis*. DAAs were therefore tested for their effectiveness to reduce biofilm growth in the presence of sub-minimal concentrations of sodium hypochlorite (NaOCl-0.031%) and Odontocide^™^ (0.25% w/v), and in the presence of Odontopaste^™^ (0.25% w/v). DAAs significantly reduced biofilm formation for all strains tested *in vitro*, while DAAs significantly reduced biofilm formation compared to LAAs. The inhibitory effect of DAAs on biofilm formation was concentration dependent. DAAs were also shown to be effective in reducing *E*. *faecalis* biofilms in the presence of Odontopaste^™^ and sub-MIC levels of NaOCl and Odontocide^™^. The results suggest that the inclusion of DAAs into current endodontic procedures may reduce *E*. *faecalis* biofilms.

## Introduction

The invasion by bacteria and their by-products into the pulp and periapical tissues respectively give rise to pulpal necrosis and apical periodontitis [1]. In fact, it is the bacterial biofilms that are the most likely etiological agent of primary and post-treatment apical periodontitis [2].

The chemo-mechanical preparation of a root canal is not sufficient to completely eliminate bacteria from the root canal. The use of an intra-canal medicament placed in-between appointments has been suggested to achieve the maximum reduction of bacterial load prior to obturation [3]. The most common reason for root canal treatment failure and the persistence of apical periodontitis is the presence of intra-radicular bacteria that had not been eliminated during endodontic therapy [2]. The use of calcium hydroxide as an intra-canal medication has been associated with periradicular healing and high antibacterial efficacy [4].

Antimicrobial agents within medicaments must be able to penetrate into the many dentinal tubules, apical deltas and accessory canals and reach a concentration that will eliminate disease causing bacteria. Calcium hydroxide has an inherently high pH and acts by damaging cytoplasmic membranes, interfering with enzymatic reactions and denaturing DNA [4, 5]. Calcium hydroxide increases the pH of the main root canal to about 12.2, however a pH of only 7.4 to 9.6 is shown to be achieved in peripheral dentine [6]. This is supported by other studies have shown that hydroxyl ions take 1–7 days to reach the outer root dentine and 3–4 weeks to reach peak pH levels [4]. Within this time, bacteria may respond by eliciting a stress response which promotes survival and persistence. One such example is an increase in biofilms.

*Enterococcus faecalis* is the most frequent species isolated from obturated root canals, with prevalence values reaching up to 90% [7–9]. Importantly its ability to penetrate deep within dentinal tubules enables the organism to escape the effects of endodontic medicaments, irrigants and instruments [6]. *E*. *faecalis* is also resistant to high pH and is extremely proficient at invading dentinal tubules where the pH increases over an extended period of time because of the buffering effect of organic matter [6]. The persistence of *E*. *faecalis* can also be attributed to the biofilm which acts as a defensive mechanism when exposed to environmental stresses [10, 11]. Thus the development of biological anti-biofilm strategies has been attracting considerable interest in endodontology [12].

Kolodkin-Gal et al. (2010) [13] demonstrated that four D-amino acids, (DAAs) D-leucine, D-methionine, D-tryptophan, and D-tyrosine were produced at late stages of biofilm growth and could disrupt axenic *Bacillus subtilis* biofilms. It was concluded that biofilm disruption resulted from the displacement of D-alanine in the peptide side chains of peptidoglycan. This is thought to trigger the release of TasA fibres from the cell wall releasing the bacteria from the biofilm. Leiman et al. (2013) [14] also investigated the mechanism by which DAAs disrupted biofilms in *B*. *subtilis* but concluded that DAAs had a toxic effect on protein synthesis which reduced the growth rate of the organism.

Although the mechanism of action of DAAs is equivocal, the ability of DAAs to act as biofilm breakers may have significant medical implications by increasing the effectiveness of antimicrobial compounds. Clinically, the use of DAAs may have application by increasing the success rate of root canal retreatment by reducing *E*. *faecalis* biofilms. Amongst the root canal medicaments currently available, Odontocide^™^ (ADM, Brisbane, Qld. Australia), contains calcium hydroxide (20% w/w) and therefore relies on high pH to eliminate viable bacteria, as well as ibuprofen (7% w/w). A different root canal medicament, Odontopaste^™^ (ADM), is used mostly because of its anti-inflammatory effect. It contains clindamycin hydrochloride (5% w/w) and triamcinolone acetonide (1% w/w). Clindamycin is effective against a broad spectrum of microbes associated with endodontic infections however *E*. *faecalis* shows significant intrinsic resistance to this antibiotic [15, 16].

The main aim of the present study was to determine if DAAs inhibit initial biofilm formation and/or reduce established biofilms produced by a number of clinical *E*. *faecalis* strains. The second aim was to determine the efficacy of DAAs in reducing *E*. *faecalis* biofilms grown under stress conditions by including the presence of sodium hypochlorite, Odontopaste^™^ and Odontocide^™^ at sub-inhibitory concentrations (sub-MIC).

## Materials and methods

### Origin, maintenance of bacterial strains and biofilm determination

Thirty-seven *E*. *faecalis* clinical strains were obtained from the Microbiology and Infectious Disease Laboratory, SA Health, South Australia (Table 1). All strains were isolated from individual patients and seven of the strains were isolated from urine while Ef42684193 was isolated from an arm abscess, Ef42657477 from phalanx tissue and the origin of Ef33212332 was unknown. Purity of *E*. *faecalis* strains were confirmed by plating on Bile aesculin agar (Oxoid, VIC, Australia) at regular intervals.

**Table 1:** *E*. *faecalis* strains supplied by the Microbiology and Infectious Disease Laboratory, SA Health, South Australia.

Ef 43730132*	Ef 33212332*	Ef 42657477*	Ef 43511662*	Ef 42684193*
Ef 42868116*	Ef 35745269*	Ef 99946846*	Ef 43730472*	Ef 33261642*
Ef 34609507P	Ef 3325660	Ef 33244403	Ef 42691219	Ef 36247218
Ef 91300766	Ef 32406104	Ef 40355126	Ef 15036	Ef 91300834
Ef 19630455	Ef 42669452U	ATCC V583	Ef 90013267	Ef 42648689
Ef 43727181	Ef 42688805	Ef 42326694	Ef 41610762	Ef 40703880
Ef 41870098	Ef 35664682	Ef 91300144	Ef 43296046	Ef 43510641
Ef 90019393	Ef 43731702			

* Strains selected for the study.

All strains were screened for their ability to produce biofilms using a modified micro-titre tray method described by Toledo-Arana et al. (2001) [17] and optimised for *E*. *faecalis* as described by Extremina et al. (2010) [18]. Briefly, each isolate was grown in Todd Hewitt Broth (THB, Oxoid, Melbourne, Vic. Australia) overnight and standardised to an OD_600nm_ of 0.5 which approximated to1X10^7^ colony forming units (CFU)/ml). 10μL of the inoculum was added to each well of a sterile flat bottom 96 well microtitre tray (Corning Inc. NY, USA). Microtitre trays were incubated at 37°C in aerobic conditions for 24 hours on an orbital mixer (Ratek. Boronia, Vic. Australia). After incubation, the bacterial suspension in each well was removed and placed into a new micro-titre tray, the optical density (λ = 570nm) of the planktonic culture was read using a microtitre plate reader (Bio-Tek PowerWave XS, Winooski Vt. USA) to confirm growth in all wells.

For the micro-titre tray containing the biofilm, all wells were rinsed with phosphate buffered saline (PBS) three times to remove non-adherent bacteria and air-dried for 1 hour at room temperature. 100μL of crystal violet solution (0.2% w/v; Oxoid) was then added to each well for 15minutes. The crystal violet was removed and the wells were rinsed again with PBS to remove excess dye. Trays were dried again for 15 minutes at room temperature before 100μL of a mixture of ethanol acetone (80:20) solution was added to each well. After 5 minutes the optical density (OD_570_ nm) of microtitre trays was read using the microtitre plate reader.

Duplicate biofilm assays were performed and all *E*. *faecalis* strains were ranked from highest to lowest biofilm producers based on the crystal violet assay.

### Effect of DAAs on the growth rate of *E*. *faecalis*

To determine whether DAAs had an inhibitory effect on the growth of each *E*. *faecalis* strain, an inoculum grown overnight in THB was standardised to give an OD_600_nm of 0.5 and 10μL was added to 8 replicate wells of a 96 well micro-titre tray containing 200μL of THB and 25mM each of D-Leu, D-Met and D-Trp and 0.25mM D-Tyr. Trays were incubated aerobically and the optical density read (560nm) periodically over 6 hours. All experiments included triplicates and controls that did not contain DAAs.

### Effect of DAAs on *E*. *faecalis* biofilms

The 10 *E*. *faecalis* strains categorised as the best biofilm producers were used to test the ability of DAAs to affect biofilm growth over a period of 24 hours, 72 hours and 144 hours. Thirty-six wells of a ninety-six well microtitre tray containing 200μL THB were inoculated with 10μL of a standardised culture (OD_600nm_ = 0.5) containing a particular *E*. *faecalis* strain. A 10μL aliquot containing D-Leu, D-Met and D-Trp was added to give a final concentration of either 0.25, 2.5 or 25mM. Due to the solubility limitations of D-Tyr, a concentration of 0.25mM was used for all experiments. For the 24 hour growth period, DAAs were added at the same time as *E*. *faecalis* inoculation, for the 72 hour biofilm growth period, DAAs were added at 24 hours after *E*. *faecalis* inoculation and for the 144 hour growth period, DAAs were added at 72 hours after *E*. *faecalis* inoculation. The rationale was to determine the effect of DAAs on initial biofilm development and on established biofilms which had grown over 24 and 72 hours.

Controls (n = 36) had DAAs omitted and other wells contained only THB (n = 24). The effect on biofilm growth was determined using the crystal violet staining protocol previously described. A reduction in optical density was considered proportional to a decrease in biofilm growth. Using the same protocol, DAAs and their cognate L-amino acids (LAAs) at a final concentration of 25mM (0.25mM, L-Tyr) were compared in their ability to reduce *E*. *faecalis* biofilms. All experiments were performed in duplicate.

### Effect on biofilms after addition of DAAs to endodontic medicaments

Minimal inhibitory concentrations (MIC) of sodium hypochlorite (NaOCl) and Odontocide^™^ (containing 20% calcium hydroxide) for all 10 *E*. *faecalis* strains were determined as previously described [11]. The MIC of NaOCl for all strains was 0.062% and 0.5% for Odontocide^™^. Biofilm assays were then performed in sub-MIC concentrations of NaOCl (0.031% v/v) and Odontocide^™^ 0.25% (w/v—pH 8.5). As none of the *E*. *faecalis* strains was sensitive to Odontopaste^™^ it was decided to use a concentration of 0.25% (w/v) Odontopaste^™^.

For each assay using a sub-MIC of NaOCl, Odontocide or Odontopaste, each agent was added simultaneously with DAAs using the same time protocol stated above. All experiments were performed in duplicate.

As with all biofilm assays, immediately following incubation, 100μL of the planktonic culture was removed and placed in another 96 well micro-titre tray, the optical density (λ = 570nm) was then measured to determine if equivalent growth had occurred during incubation in all wells.

The effect on biofilm growth was determined using the crystal violet staining protocol. A reduction in optical density was considered proportional to a decrease in biofilm growth.

### Statistical analysis

All data was analysed using three linear mixed-effects models (on day 1, 3 and 6) to examine the association between optical density and the treatment/concentration group whilst controlling for clustering on strain and treatment/concentration (i.e. clustering on plate). For all models assumptions of a linear model were only met when the logarithmic transformation of the outcome density was used. Therefore estimates and 95% confidence intervals have been exponentiated.

## Results

### Biofilm assay of *E*. *faecalis* strains

**Fig 1:**
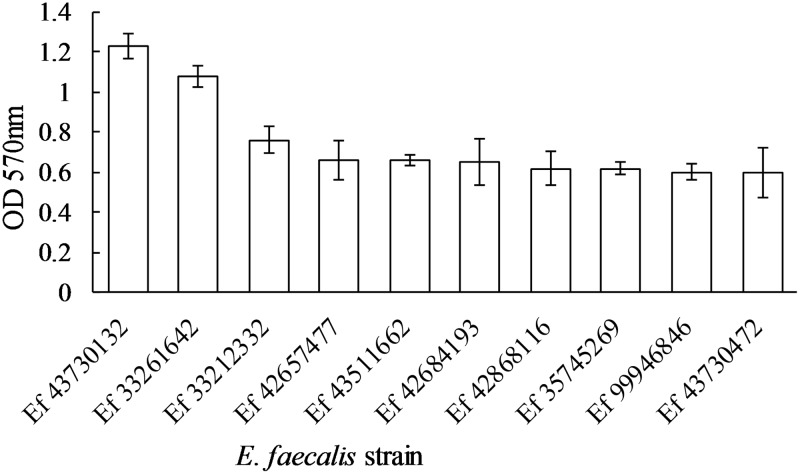
Mean optical density of each E. *faecalis strain*. Mean optical density of the ten strains used in the study. Optical density (570nm) was proportional to biofilm growth as determined by the level of crystal violet staining of the biofilm. The x-axis contains the identification of each *E*. *faecalis* strain.

Using the crystal violet staining protocol, the level of biofilm (OD_570_nm) produced by the strains is shown in Fig 1. Of the ten strains used, the lowest biofilm producers were strains Ef99946846 and Ef43730472 and these produced biofilm levels approximately 100% lower compared to the maximum (Ef43730132).

### Effect of DAAs on the growth rate of *E*. *faecalis*

**Fig 2:**
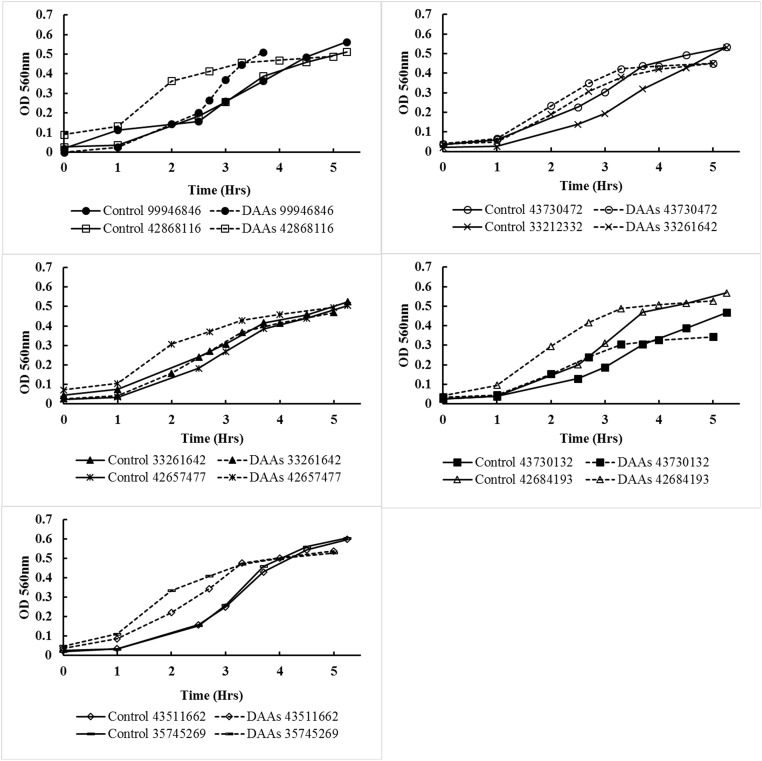
The effect of DAAs on the growth rate of *E*. *faecalis*. The growth rate as shown by optical density of each *E*. *faecalis* strain after the addition of 25mM D-Leu, D-Met and D-Trp and 0.25mM D-Tyr. DAAs were excluded from the controls.

To exclude the possibility that DAAs had a negative effect on the growth rate and therefore biofilm forming ability, 25mM D-Leu, D-Met and D-Trp and 0.25mM D-Tyr was added to the growth medium (THB) and growth was monitored over 6 hours during incubation. The addition of DAAs had no significant effect on the growth rate compared to the controls of all of the *E*. *faecalis* strains (Fig 2).

### Effect of DAAs on *E*. *faecalis* biofilms

**Fig 3:**
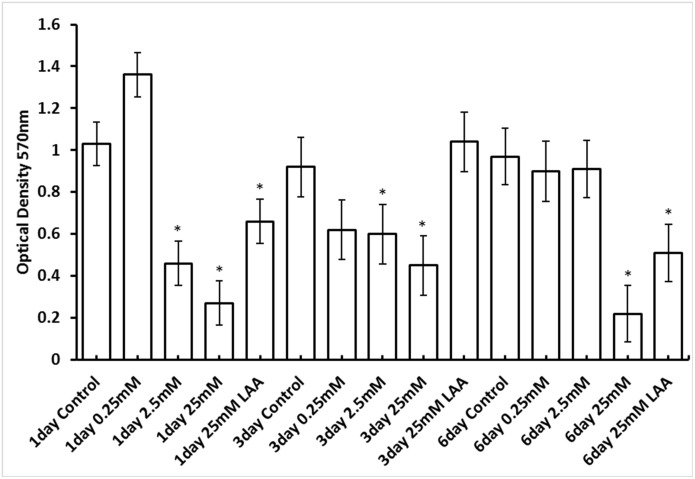
Mean optical density (570nm) of Control group (no amino acids) versus treatment group (D- or L-amino acids). Biofilm growth following staining of *E*. *faecalis* biofilms with crystal violet. For biofilms grown over 1 day, D-Leu, D-Met and D-Trp were added during inoculation of bacteria at a concentration of either 0.25, 2.5 or 25 mM. The concentration of D-Tyr was 0.25mM in all experimental groups. The same concentrations of DAAs were added at 1or 3 days for biofilms grown for a total of 3 and 6 days respectively. L-amino acids (L-Leu, L-Met and L-Trp) were also included as a control at a concentration of 25mM with L-Tyr at 0.25mM. Asterisk denotes a significant reduction (p<0.05) in biofilm compared to the control (no DAAs).

The simultaneous addition of the inoculum with 2.5mM (0.25mM Tyr) or 25mM (0.25mM Tyr) DAAs produced a significant (p < 0.0001 for both concentrations) reduction in biofilm optical density when incubated for 1 day (Fig 3). The same concentrations of DAAs produced a significant decrease in *E*. *faecalis* optical density of the biofilm when added at T = 1 day for the 3 day growth period (p = 0.0390 for concentration 2.5mM and p = 0.0007 for concentration 25mM). When the biofilm was grown over 6 days, only the addition of 25mM DAAs at T = 3 days produced a significant (p<0.0001) reduction in biofilm optical density (Fig 3).

**Fig 4:**
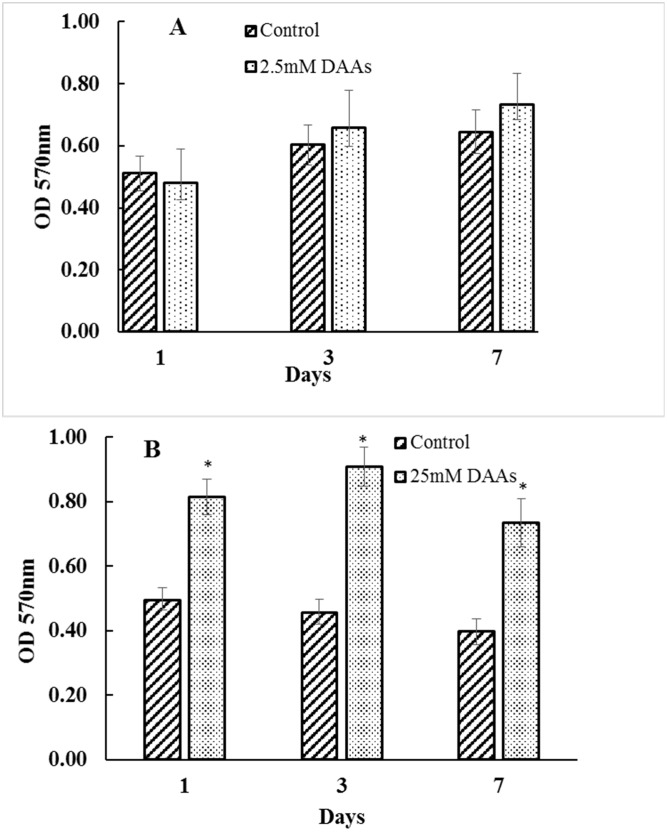
Mean optical density of the Planktonic cultures during biofilm growth. The mean optical density (570nm) of the planktonic culture removed after 1, 3 and 7 days incubation in the presence of 2.5mM (A) or 25mM (B) L-Leu, L-Met and L-Trp (0.25mM D- Tyr in both treatments). Asterisk denotes a significant reduction (p<0.05) in biofilm compared to the control (no DAAs).

To determine if DAAs inhibited growth of *E*. *faecalis* and therefore affected biofilm growth, the planktonic culture of the microtitre tray was transferred into another microtitre tray and the optical density (570nm) was recorded using a plate reader. No significant difference in culture densities were observed in the 0.25 or 2.5mM L-Leu, L-Met and L-Trp (D-Tyr = 0.25mM) concentrations although the optical density of the DAA containing cultures were higher in the latter treatment (Fig 4). The addition of 25mM D-Leu, D-Met and D-Trp (D-Tyr 0.25mM) did however produce a significant difference in the optical density of the planktonic cultures at day 1, 3 and 7.

When the DAAs were replaced with their cognate LAAs at a concentration of 25mM, (0.25mM L-Tyr) a significant decrease in biofilm growth optical density occurred at the 1 and 6 day growth periods (Fig 3) compared to the controls. However the decrease in optical density with DAAs was significantly greater than the decrease with LAAs (p < 0.0001 for T = 1 day and p = 0.0001 for T = 6 days). LAAs had no significant effect on the biofilm optical density over 3 days compared to the control (Fig 3).

### Effect on biofilms following addition of DAAs to endodontic medicaments

**Fig 5:**
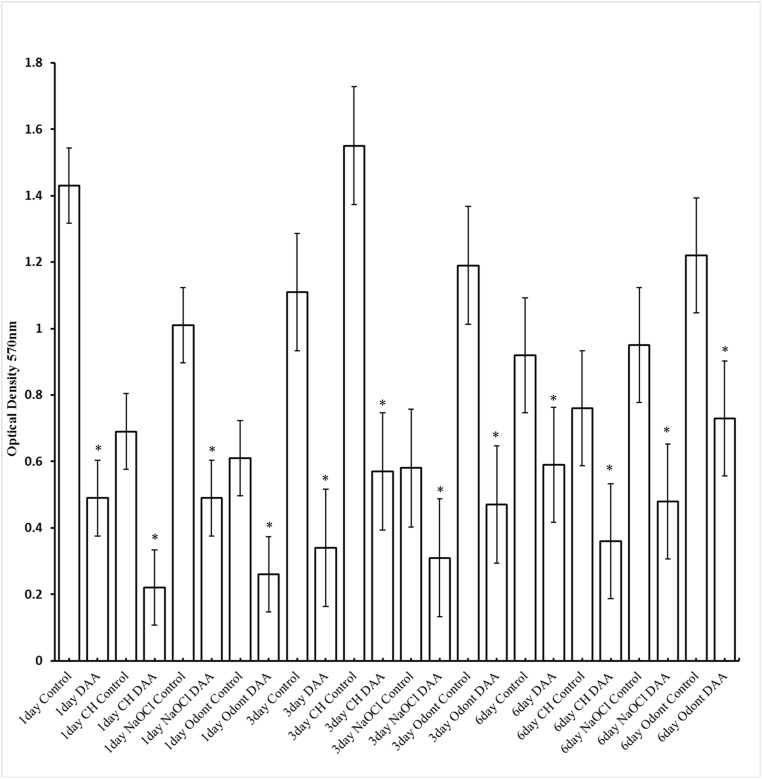
Mean density (570nm) of Control group (no D-amino acids) versus treatment groups. Biofilm growth following staining of E. faecalis biofilms with crystal violet. The medicaments Odontopaste^™^ (Odont), Odontocide^™^ (CH) and the irrigant, sodium hypochlorite (NaOCl) were added separately with D-Leu, D-Met and D-Trp (25mM) and 0.25mM D-Tyr at the time of inoculation for the 1day biofilm, then 1 day and 3 days for the 3 and 6 day biofilm respectively. Asterisk denotes a significant reduction in biofilm growth compared to controls (medicament or irrigant only).

To determine whether DAAs had an inhibitory effect on *E*. *faecalis* biofilms in the presence of commonly use endodontic medicaments and irrigants, D-Leu, D-Met and D-Trp (25mM) and 0.25mM D-Tyr were mixed with a sub-MIC concentration of NaOCl and Odontocide^™^. Odontopaste^™^ (0.25% (w/v) was also included and controls included the presence of the endodontic agent with DAAs omitted and the addition of 25mM DAAs without the agent (Fig 5).

As with the previous experiment (Fig 3) the presence of D-Leu, D-Met and D-Trp (25mM) and 0.25mM D-Tyr significantly reduced biofilm levels grown over 1, 3 and 6 days (all p < 0.0001).

When only the endodontic agent was included, the biofilm grown over 1 day was significantly reduced in the presence of sub-MIC of Odontocide^™^ NaOCl and Odontopaste^™^ (all p < 0.0001). Only NaOCl significantly reduced biofilm levels when grown over 3 days (p = 0.0074) and none of the agents significantly reduced biofilm growth over 6 days compared to the controls (Fig 5).

In the presence of Odontocide^™^, D-Leu, D-Met and D-Trp (25mM) and 0.25mM D-Tyr significantly decreased biofilm growth at 1, 3 and 6 days (p < 0.0001, < 0.0001 and < 0.00017 respectively; Fig 5) compared to the controls (Odontocide^™^ only). The addition of DAAs also produced a significant decrease when added to Odontopaste^™^ and NaOCl with the 1 day (both p <0.0001), 3 day (p = 0.0002 and 0.0102 respectively) and 6 day biofilm (p = 0.0278 and 0.0043 respectively; Fig 2).

## Discussion

It has been suggested that contemporary root-canal treatment should progress into “mechano-chemo-biological” procedures [12]. Possible strategies include weakening the microbial biofilm structure *per se* [12] so that the antimicrobial agent is more effective. The biofilm breaking effects of four DAAs (D-Leu, D-Met, D-Trp, and D-Tyr) has been demonstrated in *B*. *subtilis*, *Staphylococcus epidermidis* isolated form ocular infections [13, 19] and Li et al. (2016) [20] reported that an equimolar concentration of a mixture of DAAs acts as a biocide enhancer against a multispecies biofilm prevalent in industrial pipes. Interestingly the effect could not be reproduced using individual amino acids, so, in the present study, it was decided to only examine the anti-biofilm effect of a mixture of DAAs.

The mechanism which DAAs reduce biofilms remains elusive and several reports have suggested they affect protein synthesis, become incorporated into the cell wall in place of D-Ala or act as signal molecules which enable bacteria to adjust to a changing environment [13, 14, 21]. In the present study, it was hypothesised that DAAs could have a similar effect on biofilms produced by the endodontic pathogen, *E*. *faecalis*. The primary objectives were to determine if DAAs reduce initial biofilm development and/or reduce established biofilms and to determine if DAAs had the potential to enhance the antimicrobial action of endodontic irrigants/medicaments by acting as “biofilm breakers” thereby reducing the resistance of the *E*. *faecalis*.

Speculation remains as to whether *E*. *faecalis* can been considered a resident of the oral cavity although most consider it is more likely to be a transient organism originating from external sources. Strains of *E*. *faecalis* used in the present study were isolated from a number of non-oral sites in human infections so the results of the study may also be important for other clinical areas. Based on their ability to produce biofilms, 10 of the 37 strains were chosen to investigate the effects of DAAs on biofilm growth. Several strains produced only minimal levels of biofilm when grown over 24hours so these were excluded.

We examined the effect of DAAs on the growth rate of the *E*. *faecalis* strains used in the study and found that at the maximum concentration (25mM D-Leu, D-Met and D-Trp and 0.25mM D-Tyr) DAAs did not significantly affect the growth rate. This would assume that the DAAs did not have an inhibitory effect on protein synthesis however, further studies are required to determine the mechanism of biofilm inhibition of *E*. *faecalis* by DAAs.

Contrary to the current evidence, Sarkar & Pires (2015) [22] could not reproduce the anti-biofilm effect of DAAs at millimolar concentrations on *B*. *subtilis*, *S*. *epidermidis* or *Staphylococcus aureus*. This highlights the level of complexity in dealing with changes in DAA concentration, time of exposure/biofilm growth and the bacterial species used. We examined the effect of DAAs on initial and established biofilms and then investigated the effectiveness of the incorporation of DAAs into commonly used endodontic irrigants/medicaments. Wilson et al. (2015) [11] demonstrated that some *E*. *faecalis* strains showed significantly increased biofilm formation in the presence of sub-MIC concentrations of commonly used root canal antimicrobials/irrigants. This may be a bacterial ‘stress response’ to the presence of antimicrobial agents so it was essential to determine if DAAs could disrupt *E*. *faecalis* biofilms in environments that may also stimulate biofilm formation. Indeed, Xu et al. (2102a, 2012b) [23, 24] found that an antimicrobial stress improved biofilm disruption using DAAs.

There are numerous methods for the assessment of the ability of enterococci to produce biofilms on abiotic surfaces [25, 26]. However, the quantification of biofilms can be modified by a wide variety of test conditions, including the growth medium, the duration of growth of the bacterial inocula, the type of surface and the washing procedures [27–31]. In the present study, biofilm quantification measured the total biofilm biomass containing both live and dead cells and was optimised for *E*. *faecalis* using the protocol of Extremina et al. (2010) [18]. The microtitre tray method can achieve a large replicate sample size due to the large number of wells. Despite not accurately simulating the dentinal surface, biofilms were grown on polystyrene wells which were tissue culture treated by the manufacturer to promote cell attachment and growth. Plates were also incubated in the dark on a rotating platform to provide shear forces to aid biofilm growth.

The results demonstrate a concentration dependent reduction in biofilm growth in the presence of DAAs. At a concentration of 2.5mM and 25mM, D-Leu, D-Met and D-Trp and 0.25mM D-Tyr significantly reduced biofilms for all 10 *E*. *faecalis* strains grown over 1 and 3 days. However, only a concentration of 25mM DAAs significantly reduced biofilms grown over 6 days. The addition of DAAs at T = 0 for the 1 day biofilm was used to determine if DAAs could disrupt and reduce initial biofilm formation, addition of DAAs at T = 1 day for the 3 day biofilm and at T = 3 days for the 6 day biofilm was used to determine if DAAs could disrupt established biofilms which potentially could be more resistant to antimicrobials due to the presence of an extracellular capsule. The quantities of DAAs used in this study were greater than those used in previous studies as we intend to investigate the sustained release of DAAs over a time period consistent with endodontic treatment.

Treatment groups that showed decreased levels of biofilm also showed increase levels of planktonic cells (Fig 4). This may be explained by the inhibition or dispersion of biofilms which would be expected to increase the number of planktonic cells. Although not significantly different, 2.5mM DAA concentrations of D-Leu, D-Met, D-Trp (D-Tyr 0.25mM) showed increases in planktonic cells numbers in pre-established biofilms (3 and 7 days). Increasing the concentration of D-Leu, D-Met, D-Trp to 25mM however produced significantly more planktonic cells. We did not find significant differences when the DAA concentration was 0.25mM which is consistent with our data showing no effect on biofilm disruption at this concentration.

The addition of the cognate LAAs were also used to compare the inhibition of biofilms with DAAs. Despite their identical molecular mass and similar physical appearance, compared to the controls, (no LAAs), the inclusion of 25mM L-Leu, L-Met, L-Trp and 0.25mM L-Tyr significantly reduced levels of biofilms grown over 1 and 6 days (Fig 3). However inclusion of DAAs significantly reduced biofilm growth compared to LAAs. Kolodkin-Gal et al. (2010) [13] concluded that LAAs had no significant effect on biofilm formation by *B*. *subtilis* although concentrations of LAAs were lower (5mM) than the present study.

DAAs were also shown to be effective in disrupting and reducing *E*. *faecalis* biofilms in the presence of Odontopaste^™^ and sub minimum inhibitory concentrations of NaOCl and Odontocide^™^. When subjected to environmental stresses, biofilms use various mechanisms to promote growth and remain viable [32, 33]. The presence of only Odontopaste^™^ and sub-MIC concentrations of NaOCl and Odontocide^™^ reduced biofilm growth in the 1 day biofilm which suggests the biocides disrupted the attachment of bacteria to the surface. The biocides did not significantly reduce the optical density however when added at T = 1 day for the 3 day biofilm and at T = 3 days for the 6 day biofilm. DAAs however were able to significantly reduce and disrupt *E*. *faecalis* biofilms when added simultaneously with the biocides. Clinically, DAAs may be reduce *E*. *faecalis* biofilms where sub MIC concentrations of irrigants (e.g. NaOCl) and medicaments (e.g. Odontocide^™^) exist within the root canal system. The dentinal tubules also buffer the pH of the calcium hydroxide present in medicaments such as Odontocide^™^ by absorbing hydroxyl ions which creates an environment containing sub-lethal levels of medicament that can potentially promote biofilm growth [4, 11].

In a clinical setting, an intracanal medicament would be placed in the root canal for approximately 1–4 weeks between appointments, we hypothesise the presence of DAAs in the medicament may allow diffusion of the DAAs down the dentinal tubules to reduce biofilms and render organisms more susceptible to killing.

In conclusion, we have demonstrated that DAAs have the ability to inhibit biofilm formation as well as disrupt established biofilms produced by clinical isolates of *E*. *faecalis*. Similar results were also produced when sub-MIC levels of commonly used endodontic medicaments were present in the environment, however further research is required to determine whether DAAs may have clinical application.
